# Environmental Assessment of Solar Photo-Fenton Processes at Mild Condition in the Presence of Waste-Derived Bio-Based Substances

**DOI:** 10.3390/nano12162781

**Published:** 2022-08-13

**Authors:** Mattia Costamagna, Antonio Arques, Vanesa G. Lo-Iacono-Ferreira, Alessandra Bianco Prevot

**Affiliations:** 1 Department of Chemistry, University of Turin, 10125 Turin, Italy; 2 Departamento de Ingeniería Textil y Papelera, Universitat Politècnica de València, 03690 Alcoy, Spain; 3 Department of Engineering Projects, Universitat Politècnica de València, 03690 Alcoy, Spain

**Keywords:** photo-Fenton, life cycle assessment (LCA), bio-based substances, water treatment

## Abstract

The assessment of environmental sustainability has assumed great importance during the study and implementation of a new process, including those aimed to waste valorization and reuse. In this research, the environmental performance of the photo-Fenton processes was evaluated using the life cycle assessment (LCA) methodology. In particular, photo-Fenton conducted in mild conditions (almost neutral pH), using soluble bio-organic substances as auxiliary agents were compared with the “classic” photo-Fenton run at pH 2.8. The evaluation was carried out both, at the laboratory level and at pilot plant scale. LCA analysis shows that working in mild conditions reduces the environmental burden associated with the use of chemicals. On the other hand, the occurring drop in effectiveness significantly increases the overall impact, thus evidencing the need of considering the process as a whole.

## 1. Introduction

In recent years, there has been an increase in the interest of the scientific community on the possible valorization of bio-waste [1], in particular the organic fractions of urban bio waste (UBW). In fact, these fractions can be used both as fuels for energy production, but also as feedstock in the biorefinery sector to be converted into valuable compounds for applications in a variety of market segments [2,3,4,5,6]. In the European geographical context, these research fields find a strong response and incentive also at the political and economic level; as a matter of fact, models introduced by the concepts of circular economy and bioeconomy have been assumed as tools to reduce the climate footprint of society and other environmental burdens [7].

In recent years, the recovery of bio-based substances (BBS) from UBWs, has been reported in the literature and proposed for a wide portfolio of applications [8,9], among them the possibility of being used as chemical auxiliaries to assist the photochemical degradation of pollutants in water [10,11]. Through a structural and physico-chemical characterization, similarity between BBS and the humic substances was highlighted [3], being the photoactivity of the latter deeply studied and recognized. BBSs show a complex lignin-derived structure containing acidic and basic functional groups bonded to aromatic and aliphatic chains. Numerous publications highlight the ability of BBS to act as a direct photosensitizer (for the formation of oxidizing species in solution) or as auxiliary in photo-Fenton processes conducted in mild conditions (almost neutral pH) [12,13,14,15].

The removal of pollutants in water is a critical issue that requires careful evaluation. Specific treatments are often required to regenerate polluted effluents in order to allow their use in agriculture, industry or household [16,17]. Some of these pollutants are organic substances that are resistant to biological treatment and their degradation must be demanded to non-conventional technologies, such as the so-called Advanced Oxidation Processes (AOPs). AOPs are based on the in situ generation of strong oxidants (such as OH·, SO4·^−^), capable of oxidizing the organic compounds. A well-known and efficient method to generate these oxidants is through the photo-Fenton process [17,18]. 

Photo-Fenton reactions provide a viable and effective water remediation option due to their ability to oxidize a wide range of organics. However, the environmental implications of these treatments, over their life cycle, remain uncertain. There are scarce studies that examine the environmental footprint of photo Fenton processes [19,20]. As indicated by Pesqueira et al. [19] the main environmental hotspot is the energy consumed to make the processes work; for this reason, the treatments that exploit solar energy are more sustainable than the equivalents that require artificial irradiation sources. The use of H_2_O_2_ as an oxidant appears to be the second cause of the impacts associated with these processes, and it is emphasized that it is necessary to approach stoichiometric quantities of this oxidant to optimize the process [21]. It is interesting to note that in order to contain economic costs and environmental impacts, attempts are made to conduct the solar photo Fenton (SPF) processes in conditions of pH close to neutrality, avoiding the pre/post treatment acidification/neutralization steps. However, as highlighted by Gallego-Schmid et al. [22] processes carried out in mild conditions might be environmentally more impactful than their equivalent in an acid environment. One of the main reasons leading to this result is the high energy consumption required for the production of the required chemical auxiliaries (e.g., ethylenediamine-N,N’-disuccinic acid (EDDS), a substance used as a complexing agent in the processes at near neutral pH) [22]. As indicated by Gallego-Schmid et al., an interesting solution to reduce the environmental burden of the neutral SPF process could be the use of alternative complexing agents. The use of BBS—isolated from urban or industrial organic waste—could represent this interesting alternative and would also benefit waste valorisation, in line with the “circular economy” model, and the “waste cleaning waste” approach [10]. 

The general purpose of the present study is to evaluate, from an environmental point of view, SPF processes carried out in mild conditions in the presence of waste derived bio-based substances. In particular, the sustainability of waste valorization should not be taken for granted, but this feature remains still to be demonstrated. In the present work, the evaluation is performed by means of the Life Cycle Assessment (LCA) methodology, that enables the assessment of the environmental impacts associated with a product or a process along its entire life cycle. More specifically, the goal is to evaluate the environmental performance of the BBS as auxiliaries to run photo-Fenton process at milder pH conditions, compared to the optimum ones (i.e., pH ca. 2.8) and to identify the hotspots related to these specific treatments. 

First of all, data, at laboratory level, from the works of Bianco Prevot et al. [11] and García-Ballesteros et al. [23] on photo-Fenton processes assisted by BBS from different sourcing materials, will be revisited under the LCA perspective. Then, the LCA study will be extended to SPF experiments performed at pilot plant scale under solar irradiation and different conditions, so as to obtain more realistic data on which to base the LCA analysis. In this case the full life cycle will be taken into account, encompassing also construction, operation and decommissioning of the treatment plant.

## 2. Materials and Methods

The assessment of the environmental impacts was carried out using the software SimaPro 9 [24]. The LCA process consists of 4 phases: goal and scope definition, life cycle inventory, life cycle impact assessment (LCIA), and results interpretation.

### 2.1. Goal and Scope Definition

In the initial phase of the LCA, the system under study is identified, outlining the boundaries of the system itself; in this phase it is necessary to define the objective of the study, the functional unit and all the assumptions made. Table 1 shows a simplified scheme of the goal and scope phase for the two parts making up this work.

### 2.2. Inventory Analysis

In this phase of the LCA, all the data that constitute the input and output flows (materials, energy, emissions, waste, etc.) are collected to describe the systems analysed. Data from the Ecoinvent 3.6 database was used to model the system.

#### Laboratory Level

As previously pointed out two different BBS have been used as auxiliary agents in this study. The first, called BBS-GC, has been isolated from green compost (GC) deriving from the residues of urban public park trimming and home gardening [11,25]; the isolation process consists of a dispersion in KOH solution (at 60 °C for 4 h), followed by centrifugation; the liquid was then separated and acidified yielding to the separation of a solid phase. After centrifugation, this solid phase was recovered, washed and dried (65 °C for 24 h); a yield of ca.15% in weight was obtained.

The second BBS, called BBS-OMW, derives from olive oil mill waste (OMW) [23], through an isolation process similar to the former one: dispersion in KOH at 65 °C for 24 h, filtration and then an ultrafiltration step (molecular weight cut-off equal to 300, 150, and 50 kDa) (only the energy consumption of the pump for the filtration phase was modelled), to recover BBS in the retentate, that is ultimately dried (65 °C for 24 h); a yield of ca.40% in weight was obtained. 

The instructions provided by Piccinno et al. [26] were followed to estimate the energy consumptions for both BBS isolation processes.

The inventory data used to model the isolation processes of 1 kg of BBS are reported in Table 2.

It has been decided to model the systems with a cut-off approach [27]; the transport of the biowaste to the place—where the isolation process is performed—is considered as the “cradle” of our process. Any input and output related to the previous life of the waste have been neglected. A distance of 60 km was assumed for transporting the waste to the processing point. Since the modelling of the study was carried out by locating the system in Spain, the energy mix used is that of this country.

The environmental burdens of the photo Fenton treatment at laboratory level were tested using different BBS for the degradation of an aqueous solution of caffeine, considered a marker for anthropogenic wastewater contamination [28]. For an easier evaluation of the process degradation effectiveness, it was decided to simplify the matrix by using Milli-Q water instead of real wastewater. 

In all cases, the caffeine concentration was 5 mg/L. The time needed to degrade 90% of the pollutant and consequently the energy consumed to irradiate the system during time was determined by the disappearance profiles of the pollutant, as reported in the works of Bianco Prevot et al. [11] and García-Ballesteros et al. [23]. In the experimental conditions adopted, the time required to remove 90% of caffeine was 10 min and 15 min respectively for the experiment performed with BBS-OMW and BBS-GC. As Table 3 shows, these photo-Fenton treatments require the addition to 250 mL of the polluted solution of the following chemicals: iron salt (as catalyst), hydrogen peroxide (as oxidant), BBS (as auxiliary agent), sulfuric acid (to set the pH to 5), sodium hydroxide (to restore the original pH at the end of the process) and a light source to irradiate the system with emission in the UV-VIS range. Energy consumption is directly proportional to the time it takes to remove 90% of the initial caffeine concentration.

### 2.3. Pilot Plant Level

For a reliable and realistic assessment of the actual environmental performance of solar photo Fenton process, the second part of the present work has been based on data obtained from a real pilot plant. This plant—built to test technology for wastewater treatment—is described in the following sections.

Caffeine degradation tests were carried out using 2 concentrations: 1 mg/L and 50 mg/L, different from what previously observed at the laboratory level. This choice was made to collect information on a wider range of possible contaminant concentration with the aim of testing and identifying possible and more concrete behavior differences (and therefore associated environmental impacts) of the tested process.

The operating conditions of the solar photo Fenton processes considered can be summarized as follows:

- solar photo-Fenton at pH 2.8, caffeine concentration 1 mg/L
- solar photo-Fenton at pH 2.8, caffeine concentration 50 mg/L
- solar photo-Fenton at pH 5.0, caffeine concentration 1 mg/L
- solar photo-Fenton at pH 5.0 with BBS-OMW, and caffeine concentration 1 mg/L
- solar photo-Fenton at pH 5. with BBS-OMW, caffeine concentration 50 mg/L and increase of the reactant’s concentration

The experiment ran at pH 5.0 with BBS-OMW and caffeine at a concentration of 1 mg/L, only resulted in a slight caffeine degradation and therefore it was not included in the LCA analysis. This low efficiency could be attributed to the competition between caffeine and BBS-OMW for the reactive species generated, BBS-OMW being ten times more concentrated than caffeine.

**Figure 1:**
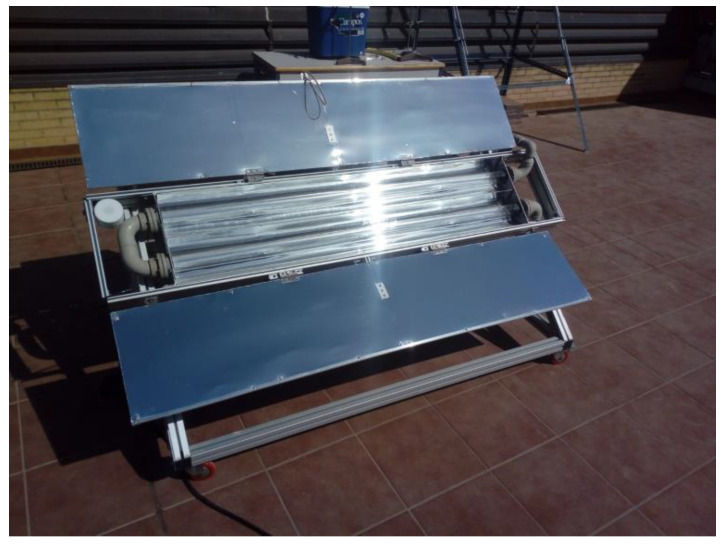
A photo of the pilot plant.

The processes tested at semi-industrial level were carried out in a solar photoreactor Solardetox Acadus-2015 (Ecosystem SA) (Figure 1), designed for decontamination of wastewaters, based on compound parabolic collector technology, CPC [29].

The plant consists of two borosilicate tubes (diameter 50 mm, thickness 2.5 mm), through which the solution to be treated flows. Two aluminium parabolic mirrors concentrate the sunlight in the axis of each tube. The total surface of the photoreactor is 0.45 m^2^, with an irradiated volume of 4.5 L. The surface is tilted 40° with the horizon and left in a sunny place with a southern orientation. The solution is circulated inside the reactor by means of a pump. The reactor is equipped with a radiometer (Acadus 85-PLS), which measured the received UV-A radiation; the radiometer contribution, however, was neglected in LCA modelling. The inventory data relative to the pilot plant are reported in Table 4.

It was assumed that the system was manufactured in Barcelona and that the transport to its final destination (Alcoy) was done by truck. The lifespan of the entire plant is estimated to be 20 years; the borosilicate glass tubes and the pump, on the other hand, must be replaced after 10 years of work (inputs already considered in the inventory reported in Table 4). To model the decommissioning phase of the plant, it was assumed that the main materials (steel, aluminum, glass and polypropylene) are recycled.

The system was modelled to treat 1 m^3^ of water per day in order to obtain more reliable data. The sizing of the pilot plant was made taking into account the m^2^ of surface needed for the treatment of the desired volume of water in one day. In fact, this value can be obtained knowing that:

The average radiation received in a day, in the Alcoy region, is 18 W/m^2^, equivalent to 1555.2 kJ/m^2^. 

The accumulated radiation (AR) necessary for water treatment is measured by means of the radiometer, during the caffeine degradation tests; this value corresponds to the amount of solar energy necessary for the complete degradation of the contaminant.

By applying a simple proportion (1)

1555.2 (kJ):1 (m^2^) = AR (kJ):X (m^2^) (1)

it is possible to calculate the m^2^ of reactor surface that would be necessary to treat 1 m^3^ of water per day in the different conditions tested. The number of equivalent pilot plants to obtain the desired surface is easily determined, knowing the real m^2^ of the reactor area. Table 5 shows the accumulated radiation, the required surface and the corresponding number of equivalent pilot plants, necessary for the caffeine degradation based on the results of caffeine degradation test in the different experimental conditions.

The degradation efficiency is much higher at pH 2.8, both for low and high concentrations of caffeine. Hence, these processes require a smaller surface area for capturing sunlight and therefore a small-sized plant is sufficient. It should be noted that the process at pH 2.8 with a caffeine concentration of 1 mg/L required a surface lower than that of one plant; however, it was decided to keep the minimum value of 1 plant in the modeling.

Since the functional unit refers to the process completed in 1 working day, the contributions of an equivalent pilot plant have been normalized assuming that during its life (20 years) it can be used for 4400 working days (see Table 6).

The modelling of the use phase was carried out on the basis of the input-output data determined running the photo Fenton experiments. The energy demand of the pump (0.44 kWh/day, per pilot plant), which allows the circulation of the solution under treatment inside the system, was also taken into account.

The experimental conditions were set as follows: the pH was adjusted to the desired value with H_2_SO_4_; Fe^3+^ was introduced as FeCl_3_·6H_2_O; at the end of the process NaOH was added to restore the initial pH. It should be noted that these inputs, once the degradation process is over, will remain in the water, as their concentration is very low they do not constitute a reason for damage to human health and do not exceed any law parameter relating to water. For these reasons, in the LCA analysis, it was decided not to model the emission of these substances in water. A similar approach can be applied to BBS; in this case, however, it should be emphasized that, up to now, the data to adequately assess the environmental impacts, in the LCA analysis, would be lacking. 

The inventory values for each of the four processes, with the number of equivalent pilot plants considered and the energy demand are shown in Table 6.

As Table 6 shows, the inputs of oxidant and catalyst for the process with 50 mg/L of caffeine, at pH 5.0, are higher than the others; this is because the degradation process in this condition is less effective, and a greater amount of chemicals is required to make it happen.

## 3. Results

The environmental impacts have been estimated according to the EF 3.0 method [30].

The impacts are reported at midpoint level and 8 categories have been considered: climate change (CC), ozone depletion (OD), human toxicity-non-cancer (HT-nc), human toxicity-cancer (HT-c), acidification (A), freshwater eutrophication (FE), resource use, fossils (RU-f) and resource use, minerals and metals (RU-mm). It should be noted that according to the Technical Report by JRC [30] CC and OD are recommended and considered satisfactory impact categories; A and FE are recommended although they need some improvement; HT-nc, HT-c, RU-f and RU-mm falls into the impact categories group of recommended, but to be applied with caution. First, the results obtained when considering photo-Fenton processes run at laboratory scale are discussed, followed by the ones obtained running the photo-Fenton treatment at pilot plant scale.

The impacts associated with the isolation of 1 kg of bio-based substances, from oil mill waste (BBS-OMW) and green compost (BBS-GC), are reported in Table 7. BBS-OMW shows higher impacts than BBS-GC for all categories, except for OD, although differences are, in general, low.

The impact for the isolation of BBS-OMW is mainly attributable to the considerable use of potassium hydroxide, necessary to create the basic conditions that allow the extraction of this substance from the native matrix; it should be noted that this input, mainly generated by the BBS washing step, could easily be reduced by analyzing the quality of the isolated product at different washing steps. Instead, the impacts of the isolation process from green compost are split between energy consumption and the use of sodium hydroxide. In this case it is difficult to hypothesize a possible improvement of the extraction process to further reduce environmental impacts.

Regarding climate change, 1 kg of BBS obtained from oil mill waste produced 1.50 kg CO_2_ eq. and 1.34 kg CO_2_ eq. if BBS was isolated from green compost. Gallego-Schmid et al. (2019) analyzed the environmental performance of different photo Fenton processes, including those carried out at neutral pH using EDDS as a complexing agent. They estimated an impact of 5.06 kg CO_2_ eq per kg of substances and it appears that the complexing agent was the cause of about 60% of the total impact. Comparing both data, production of BBSs releases lower amounts of CO_2_ eq. Although comparing the efficiency of EDDS and BBS as auxiliaries for photo-Fenton, the higher sustainability of BBS is an advantage that should be considered in any case.

### 3.1. Results at Laboratory Level

Table 8 and Table 9 show the impact of photo Fenton processes, in mild conditions, performed to treat a solution of 250 mL of water containing caffeine. As reported in the Inventory section, the tests were done at laboratory level and using the two BBSs as complexing agents. In both tables a heat map introduces, for the values of each impact category, a color gradient that marks the results from red (major impacts) to green (more limited impacts).

Table 8 shows the environmental burden of a process that uses BBS obtained from olive oil mill waste.

From light green to dark green: from low to very low impact. The main impacts are associated with the consumption of electricity, used to irradiate the system in order to allow the degradation of caffeine. It can be seen that the second cause of impacts is the oxidant (hydrogen peroxide), followed by BBS. Working at pH 5 allows to limit the quantities of acid and base added pre and post process and consequently to cut down the respective environmental impacts.

Similarly, Table 9 shows the impact of the process performed using BBS isolated from green compost.

Also in this case, the energy consumption is responsible for the greatest impacts, followed by hydrogen peroxide; BBS-GC appears to be the third cause of impact for the categories: CC, OD, A and RU-f; on the other hand, for the HT-nc, HT-c, FE and RU-mm categories, the use of iron sulfate causes slightly greater impacts than BBS.

As shown in both processes, energy is the major cause of the impacts: around 99% of total impact is generated by the consumed electricity, for all the considered categories. For this reason, if the two photo Fenton processes are compared, it appears that the one conducted using BBS-OMW, given their higher efficiency and therefore the shorter time to degrade the pollutant, shows lower environmental impacts than the process carried out with BBS-GC. However, it should be emphasized that the first process uses a larger amount of oxidant (see Table 3), which is most likely the real responsible for its best performance. Therefore, the two types of BBS can be considered equivalent, both from the point of view of the effectiveness in assisting photo Fenton processes, and as regards the environmental impacts generated during their production.

### 3.2. Results at Pilot Plant Level

The LCIA results relating to the pilot plant and the SPF processes are shown below. Table 10 shows the environmental impacts generated by production of the pilot plant (the materials subject to replacement over time were also considered).

The main impacts, for all the categories, are associated with the metals used for the structure of the plant, that are steel and aluminum.

Investigating more in detail the impacts associated with the pumps, it appears that both for the HT-nc, FE and RU-mm categories the cause is due to the limited amount of copper present in some parts of these devices. The impacts generated by the use of borosilicate glass are mainly generated by the heat and energy required during their production.

**Figure 2:**
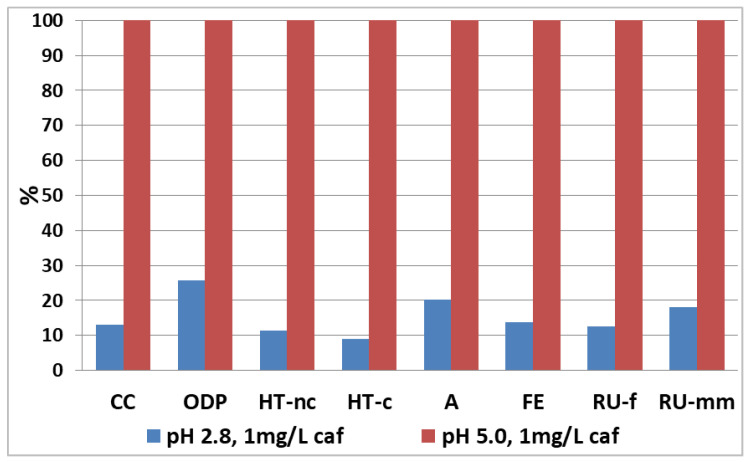
Comparison of the photo Fenton option for the degradation process of 1 m^3^ of water per day (caffeine concentration of 1 mg/L). The values are reported grouped for impact category and as a percentage, assigning 100% to the highest impact for each category analyzed.

**Figure 3:**
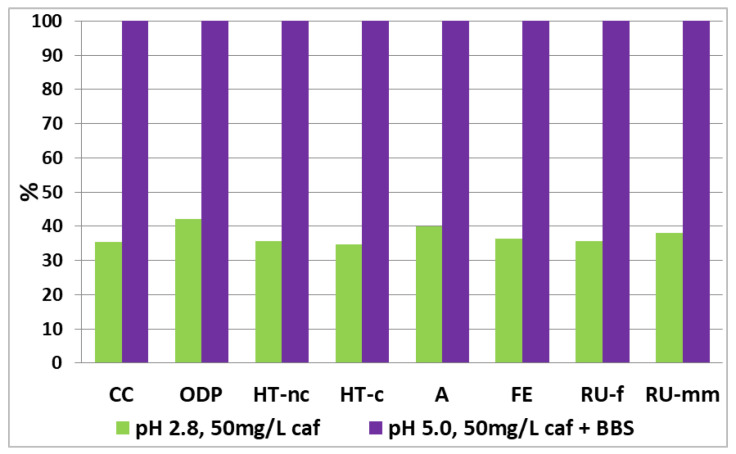
A comparison of the photo Fenton option for the degradation process of 1 m^3^ of water per day (caffeine concentration of 50 mg/L). The values are reported grouped for impact category and as a percentage, assigning 100% to the greatest impact for each category analyzed.

The results related to the SPF treatments of a solution of water containing 1 mg/L and 50 mg/L of caffeine are shown in Figure 2 and Figure 3, respectively. The impacts take into account the contribution of both the chemical process and the pilot plant itself (plant sized according to what is reported in Table 6). The results are reported in percentages, assigning the value of 100% to the highest impact for each impact category.

It can be observed how, both at 1 mg/L and 50 mg/L of caffeine, the processes at pH 5.0, have greater impacts for each category when compared to processes at acidic pH. As already reported, the process at pH 5.0, for the degradation of a 1 mg/L caffeine solution, was carried out without the use of BBS as a complexing agent; in fact, in a previous experiment it was observed how at this concentration of pollutant the BBS suppress the degradation process.

The comparison highlights a clear better environmental performance for the processes conducted at acidic pH; although in these conditions it is necessary a side phase to restore the pH through the addition of sodium hydroxide, this step does not have a considerable influence on the overall environmental impact.

However, it can be observed that the difference in impacts between acidic and near neutral pH is less marked when BBS is used to treat higher concentrations of pollutants; in this case the contribution of BBS to improve the degradation efficiency is evident, compared to the case at 1 mg/L where the pH 5.0 process was clearly inefficient (see also Table 5).

For a better understanding of the individual contribution of the impacts every single process must be analyzed separately. For this reason, the individual LCIA results of the treatment of 1 m^3^ of water carried out in the various conditions tested are reported in the Supplementary Information (Tables S3–S5).

It can be observed that the processes carried out at pH 5.0—caffeine 1 mg/L, pH 2.8 and pH 5.0—caffeine 50 mg/L show a common trend of impacts (albeit with a difference in terms of values): for all categories, with the exceptions of HT-nc, HT-c and RU-mm, the main cause of impact is the consumption of electricity; on the other hand, for the three categories just mentioned, the main cause of the respective impacts is attributable to the plant. These results derive from a limited efficiency of the degradation systems, which need to absorb more solar radiation to complete the degradation of the pollutant, what results in larger plants (i.e., larger surface exposed to the sun) and higher energy consumption to circulate the solution through the plant.

As already mentioned, the impact generated by the life cycle of the plant is the main cause of environmental burdens as regards the human toxicity (cancer and non) and resource use (minerals and metals) categories and these results are common for all the processes analyzed, therefore—with the exception of the RU-mm category—also for the process at pH 2.8 and caffeine 1 mg/L. It should be noted that these results were obtained despite the values reported taking into account the credits for the recovery of materials (steel, aluminum, etc.) at the end of the plant’s life.

As regards the degradation process itself (chemicals), it can be observed how its contribution is appreciable only at pH 2.8 and 1 mg/L of caffeine (Table S3). In fact, in this situation the dimensions of the plant and energy consumption are contained and the impacts due to the use of chemical substances are more influential on the final impacts. Working under these conditions it is noted that the use of sodium hydroxide and sulfuric acid causes the highest impacts for the OD, A and RU-mm categories respectively. For the process at pH 2.8—caffeine 1 mg/L, there is also a certain importance of the impacts associated with hydrogen peroxide, which for the categories climate change, human toxicity—cancer, eutrophication and resource use (fossils), is the second main cause of impact. 

Obviously, if analyzed in detail, the processes carried out at pH 5.0 show reduced impacts as regards the contributions of sulfuric acid and sodium hydroxide, given their use in less or no quantity. Overall, however, this reduction is very minimal compared to the total impact of each category.

Unlike what Gallego-Schmid et al. [22] observed the contribution of the complexing agents to the overall impacts is rather limited and secondary with respect to the use of hydrogen peroxide; in this case the main problem, which worsens environmental performance, is the low degradation efficiency of the process performed in such conditions.

## 4. Conclusions

Based on the results obtained in this study it can pointed out that if it is not possible to obtain a degradation efficiency similar to that of the processes conducted at acid pH, working in milder conditions (with a reduction in the intake of chemicals and the use of environmentally sustainable complexing agents) does not automatically translate into a real improvement of the impacts of the SPF processes. 

More in details, in this specific case, to ensure that the impacts generated, working at pilot plant scale, by the process at pH 5.0 are comparable or less than those of the process at pH 2.8, it would be necessary to increase the efficiency of the process at pH 5.0 by accelerating the degradation rate by a factor equal to 3.5 times compared to current value. Such an improvement would make it possible to reduce the size of the plant itself (as the volume of treatable water increases in a given time) and the energy demand. Moreover, the possibility of obtaining auxiliary agents from organic waste (such as BBS) with limited environmental impacts encourages further efforts in this direction. In fact, if scientific research will be able to obtain BBS capable of forming photoactive iron complexes and therefore to improve the efficiency of processes at near neutral pH, then a significant step toward more sustainable SPF will be done.

As a future perspective it could certainly be interesting to integrate this type of study by enriching the LCA modelling with the possible release into water of by-products and reaction intermediates, that could generate effects on the different impact categories considered.

## Figures and Tables

**Table 1:** A goal and scope scheme for the two parts of the study.

Category	Laboratory Level	Pilot Plant Level
**Goal**	To identify the main environmental hotspots of near neutral pH photo-Fenton process and to analyse in detail the environmental behaviour and performance of two waste derived BBS used as auxiliary agents: green compost-derived and olive oil mill waste-derived BBS.	To evaluate the effect of performing, at different pH (pH 2.8, pH 5.0), solar photo-Fenton process, using waste derived BBS as auxiliary agent.
**Functional unit**	The removal of 90% caffeine from 250 mL of Milli-Q water	Treatment of 1 m^3^ of water contaminated with caffeine per day.
**System boundaries**	The system was modelled considering: (i) the production of each chemical reagent (H_2_SO_4_, H_2_O_2_, etc.); (ii) the isolation process to obtain the BBS; (iii) the degradation process itself. (The infrastructure and equipment used during the experimental analysis were excluded from the calculation).	Cradle-to-grave approach, encompassing the construction, the use for degradation processes and the decommissioning of the treatment plant.
**Experimental conditions**	See Supplementary Information—Table S1.	See Supplementary Information—Table S2.

**Table 2:** Inventory data for the isolation processes of 1 kg of BBS from different sources (GC: green compost; OMW: oil mill waste).

Input	BBS-GC	BBS-OMW
Transport (tkm)	0.40	0.15
NaOH (g)	133	/
KOH (g)	/	617
Water (kg)	37	110
HCl (g)	50	/
Heat (extraction) (kJ)	6084	2306
Electricity (filtration) (kWh)	/	0.036
Electricity (centrifugation) (kWh)	0.9	/
Heat (drying) (kJ)	608	608
Process output (kg)	1	1

**Table 3:** Inventory of the input for the treatments of 250 mL of Milli-Q water contaminated with caffeine (5 mg/L), in the presence of different complexing agents: BBS-GC and BBS-OMW.

Input	Unit	Photo Fenton with BBS-OMW	Photo Fenton with BBS-GC
FeSO_4_	mg	6.2	6.2
H_2_O_2_	mg	15	6.25
H_2_SO_4_	mg	0.1	0.1
NaOH	mg	0.09	0.09
BBS	mg	2.5	2.43
Electricity	kWh	0.05	0.075

**Table 4:** Inventory data for the pilot plant.

Input	Amount	Unit
Stainless steel	55	kg
Aluminium	9	kg
Borosilicate glass	5.57	kg
Anodizing process	2	m^2^
Polypropylene (PP)	7	kg
Pumps	2	items
Transport	36	tkm

**Table 5:** The accumulated radiation, the required surface and the corresponding number of equivalent pilot plants, necessary for caffeine degradation. (*) Even if a smaller plant would be sufficient, it was decided to keep the value of 1 pilot plant in the modeling.

Parameter	1 mg/L Caffeine		50 mg/L Caffeine	
	pH 2.8	pH 5.0	pH 2.8	pH 5.0
**Accumulated radiation (kJ)**	90	11,271	5713	16,635
**Required surface (m^2^)**	0.058	7.25	3.67	10.7
**N° of pilot plants equivalent**	1 *	16.11	8.16	23.77

**Table 6:** Inventory data for the use phase: the treatment of 1 m^3^ of contaminated water, with the 4 solar photo Fenton processes considered. Data are grouped for caffeine concentration and working pH.

Parameter	1 mg/L Caffeine		50 mg/L Caffeine	
	pH 2.8	pH 5.0	pH 2.8	pH 5.0
H_2_SO_4_ (g)	284.88	174.66	270.47	220.45
H_2_O_2_ (g)	100.90	100.90	100.90	303.03
FeCl_3_·6H_2_O (g)	20.00	20.00	20.00	50.00
NaOH (g)	56.80	/	57.60	18.00
BBS-OMW (g)	/	/	/	30
Transport of chemicals (tkm)	0.05	0.05	0.05	0.05
N° of pilot plants equivalent normalized	2.3 × 10^−5^	0.0037	0.0019	0.0054
Electricity (kWh)	0.44	7.08	3.59	10.45

**Table 7:** Impact at midpoint level for the production process of 1 kg of BBS.

Impact Category	Unit	BBS-OMW	BBS-GC
Climate change (CC)	kg CO_2_ eq	1.498	1.338
Ozone depletion (OD)	kg CFC11 eq	1.75 × 10^−7^	2.84 × 10^−7^
Human toxicity, non-cancer (HT-nc)	CTUh	4.351 × 10^−8^	1.31 × 10^−8^
Human toxicity, cancer (HT-c)	CTUh	7.85 × 10^−10^	4.85 × 10^−10^
Acidification (A)	mol H^+^ eq	7.49 × 10^−3^	6.62 × 10^−3^
Eutrophication, freshwater (FE)	kg P eq	7.94 × 10^−4^	3.29 × 10^−4^
Resource use, fossils (RU-f)	MJ	24.678	22.361
Resource use, minerals and metals (RU-mm)	kg Sb eq	3.803 × 10^−5^	1.76 × 10^−5^

**Table 8:** LCIA results for the photo Fenton process, at laboratory level: treatment of 250 mL of water contaminated with 5 mg/L of caffeine, at pH 5.0, using BBS-OMW as complexing agent.

Heading	Total	FeSO_4_	H_2_O_2_	H_2_SO_4_	NaOH	BBS-OMW	Electricity
**CC** **kg CO_2_ eq**	1.69 × 10^−2^	1.07 × 10^−6^	1.77 × 10^−5^	1.13 × 10^−8^	1.20 × 10^−7^	3.75 × 10^−6^	1.69 × 10^−2^
**OD** **kg CFC-11 eq**	1.83 × 10^−9^	1.08 × 10^−13^	2.15 × 10^−12^	1.35 × 10^−15^	7.19 × 10^−14^	4.37 × 10^−13^	1.82 × 10^−9^
**HT-nc** **CTUh**	2.55 × 10^−10^	4.53 × 10^−14^	2.12 × 10^−13^	5.43 × 10^−16^	2.44 × 10^−15^	1.09 × 10^−13^	2.55 × 10^−10^
**HT-c** **CTUh**	7.91 × 10^−12^	1.60 × 10^−15^	4.40 × 10^−14^	1.70 × 10^−17^	6.36 × 10^−17^	1.96 × 10^−15^	7.86 × 10^−12^
**A** **mol H^+^ eq**	1.50 × 10^−4^	8.48 × 10^−9^	7.14 × 10^−8^	1.12 × 10^−9^	6.95 × 10^−10^	1.87 × 10^−8^	1.50 × 10^−4^
**FE** **kg P eq**	7.05 × 10^−6^	1.07 × 10^−9^	6.55 × 10^−9^	6.43 × 10^−12^	6.16 × 10^−11^	1.98 × 10^−9^	7.04 × 10^−6^
**RU-f** **MJ**	3.87 × 10^−1^	1.87 × 10^−5^	3.07 × 10^−4^	4.37 × 10^−7^	1.51 × 10^−6^	6.17 × 10^−5^	3.87 × 10^−1^
**RU-mm** **kg Sb eq**	1.37 × 10^−7^	1.06 × 10^−10^	3.27 × 10^−10^	1.51 × 10^−12^	2.74 × 10^−12^	9.51 × 10^−11^	1.36 × 10^−7^

Red: high impact. Yellow-Orange: medium impact. From light green to dark green: from low to very low impact.

**Table 9:** LCIA results for the photo Fenton process, at laboratory level: treatment of 250 mL of water contaminated with 5 mg/L of caffeine, at pH 5.0, using BBS-GC as complexing agent.

Impact Category (and Unit)	Total	FeSO_4_	H_2_O_2_	H_2_SO_4_	NaOH	BBS-GC	Electricity
**CC** **kg CO_2_ eq**	2.53 × 10^−2^	1.07 × 10^−6^	7.36 × 10^−6^	1.13 × 10^−8^	1.20 × 10^−7^	3.25 × 10^−6^	2.53 × 10^−2^
**OD** **kg CFC-11 eq**	2.74 × 10^−9^	1.08 × 10^−13^	8.94 × 10^−13^	1.35 × 10^−15^	7.19 × 10^−14^	6.90 × 10^−13^	2.73 × 10^−9^
**HT-nc** **CTUh**	3.83 × 10^−10^	4.53 × 10^−14^	8.82 × 10^−14^	5.43 × 10^−16^	2.44 × 10^−15^	3.18 × 10^−14^	3.83 × 10^−10^
**HT-c** **CTUh**	1.18 × 10^−11^	1.60 × 10^−15^	1.83 × 10^−14^	1.70 × 10^−17^	6.36 × 10^−17^	1.18 × 10^−15^	1.18 × 10^−11^
**A** **mol H^+^ eq**	2.25 × 10^−4^	8.48 × 10^−9^	2.97 × 10^−8^	1.12 × 10^−9^	6.95 × 10^−10^	1.61 × 10^−8^	2.25 × 10^−4^
**FE** **kg P eq**	1.06 × 10^−5^	1.07 × 10^−9^	2.73 × 10^−9^	6.43 × 10^−12^	6.16 × 10^−11^	7.99 × 10^−10^	1.06 × 10^−5^
**RU-f** **MJ**	5.81 × 10^−1^	1.87 × 10^−5^	1.28 × 10^−4^	4.37 × 10^−7^	1.51 × 10^−6^	5.43 × 10^−5^	5.81 × 10^−1^
**RU-mm** **kg Sb eq**	2.05 × 10^−7^	1.06 × 10^−10^	1.36 × 10^−10^	1.51 × 10^−12^	2.74 × 10^−12^	4.28 × 10^−11^	2.05 × 10^−7^

Red: high impact. Yellow-Orange: medium impact. From light green to dark green: from low to very low impact.

**Table 10:** Impact at midpoint level for the pilot plant analyzed.

	Total	Steel	Aluminum	Boro- Silicate Glass	Polypropylene	Anodi- Zing Process	Pumps	Transport
**CC** **kg CO_2_ eq**	409.71	248.39	89.81	13.15	16.67	6.65	16.55	18.50
**OD** **kg CFC-11 eq**	2.68 × 10^−5^	1.26 × 10^−5^	7.42 × 10^−6^	8.81 × 10^−7^	3.40 × 10^−7^	7.05 × 10^−7^	9.57 × 10^−7^	3.96 × 10^−6^
**HT-nc** **CTUh**	2.01 × 10^−5^	1.18 × 10^−5^	3.25 × 10^−6^	1.73 × 10^−7^	1.19 × 10^−7^	9.74 × 10^−8^	4.45 × 10^−6^	2.50 × 10^−7^
**HT-c** **CTUh**	4.60 × 10^−6^	4.14 × 10^−6^	2.17 × 10^−7^	7.95 × 10^−9^	3.84 × 10^−9^	6.44 × 10^−9^	2.23 × 10^−7^	8.69 × 10^−9^
**A** **mol H^+^ eq**	2.88	1.60	0.65	0.11	0.07	0.04	0.36	0.05
**FE** **kg P eq**	1.87 × 10^−1^	9.42 × 10^−2^	4.08 × 10^−2^	4.21 × 10^−3^	2.95 × 10^−3^	5.25 × 10^−3^	3.80 × 10^−2^	1.95 × 10^−3^
**RU-f** **MJ**	5005.53	2642.93	1106.73	158.49	527.55	115.40	180.49	273.94
**RU-mm** **kg Sb eq**	1.47 × 10^−2^	9.11 × 10^−3^	3.39 × 10^−4^	1.94 × 10^−3^	1.55 × 10^−4^	7.23 × 10^−5^	2.14 × 10^−3^	9.10 × 10^−4^

Red: high impact. Yellow-Orange: medium impact. From light green to dark green: from low to very low impact.

## Data Availability

Not applicable.

## Supplementary Materials

The following supporting information can be downloaded at: https://www.mdpi.com/article/10.3390/nano12162781/s1, Table S1: Experimental conditions for the two processes performed at laboratory level. Table S2: Experimental conditions for SPF processes performed with the pilot plant. Table S3: LCIA results for the degradation process of 1 m^3^ of water contaminated with 1 mg/L of caffeine, at pH 2.8. Table S4: LCIA results for the degradation process of 1 m^3^ of water contaminated with 1 mg/L of caffeine, at pH 5. Table S5: LCIA results for the degradation process of 1 m^3^ of water contaminated with 50 mg/L of caffeine, at pH 2.8.
