# Supplementary material for: Environmental Assessment of Solar Photo-Fenton Processes at Mild Condition in the Presence of Waste-Derived Bio-Based Substances
Source: Nanomaterials (Basel). 2022 Aug 13;12(16):2781. doi: 10.3390/nano12162781 (PMC9416024; doi:10.3390/nano12162781)
Supplement: Supplementary file 1 [file nanomaterials-12-02781-s001.zip › nanomaterials-1817485-supplementary.pdf]

Supplementary Material

Environmental Assessment of Solar Photo-Fenton Processes at Mild Condition in the Presence of Waste-Derived Bio-Based Substances

Mattia Costamagna [1], Antonio Arques [2], Vanesa G. Lo-Iacono-Ferreira [3] and Alessandra Bianco Prevot [1,*]

[1] Department of Chemistry, University of Turin, 10125 Turin, Italy
[2] Departamento de Ingeniería Textil y Papelera, Universitat Politècnica de València, 03690 Alcoy, Spain
[3] Department of Engineering Projects, Universitat Politècnica de València, 03690 Alcoy, Spain
* Correspondence: alessandra.biancoprevot@unito.it

Table S1. Experimental conditions for the two processes performed at laboratory level.

Variables	Process with BBS-GC	Process with BBS-OMW
Volume of the solution	0.250 L	0.250 L
Caffeine concentration	5 mg/L	5 mg/L
pH	5	5
Fe(II) concentration	5 mg/L	5 mg/L
H_2O_2 concentration	25.5 mg/L	60 mg/L
BBS concentration	9.7 mg/L	10 mg/L
Lamp	Xenon lamp (300 W)	Xenon lamp (300 W)

Table S2. Experimental conditions for SPF processes performed with the pilot plant.

Variables	Processes for the degradation of 1 mg/L caffeine			Processes for the degradation of 50 mg/L caffeine	
pH	2.8	5.0	5.0	2.8	5.0
Volume of the solution	1 m^3	1 m^3	1 m^3	1 m^3	1 m^3
Caffeine concentration	1 mg/L	1 mg/L	1 mg/L	50 mg/L	50 mg/L
Fe(II) concentration	4 mg/L	4 mg/L	4 mg/L	4 mg/L	10 mg/L
H_2O_2 concentration	100 mg/L	100 mg/L	100 mg/L	100 mg/L	300 mg/L
BBS concentration	/	/	20 mg/L	/	30 mg/L

In the next tables a heat map introduces, for the values of each impact category, a colour gradient that marks the results from red (major impacts) to green (more limited impacts).

Table S3. LCIA results for the degradation process of 1 m^3 of water contaminated with 1 mg/L of caffeine, at pH 2.8.

pH 2.8 - 1 mg/L of caffeine								
**Heading**	**Total**	**Plant**	**H_2SO_4**	**NaOH**	**$FeCl_3$**	**H_2O_2**	**Transport chemicals**	**Electricity**
**CC kg CO_2 eq**	3.83×10^{-1}	2.63×10^{-2}	2.51×10^{-2}	4.74×10^{-2}	8.54×10^{-3}	1.16×10^{-1}	1.07×10^{-2}	1.48×10^{-1}
**OD kg CFC-11 eq**	8.71×10^{-8}	4.06×10^{-9}	2.24×10^{-9}	4.54×10^{-8}	3.13×10^{-9}	1.39×10^{-8}	2.37×10^{-9}	1.60×10^{-8}
**HT-nc CTUh**	9.71×10^{-9}	3.24×10^{-9}	1.30×10^{-9}	1.02×10^{-9}	3.88×10^{-10}	1.39×10^{-9}	1.37×10^{-10}	2.25×10^{-9}
**HT-c CTUh**	1.46×10^{-9}	1.01×10^{-9}	4.57×10^{-11}	3.19×10^{-11}	1.15×10^{-11}	2.95×10^{-10}	4.14×10^{-12}	6.92×10^{-11}
**A mol H+ eq**	5.72×10^{-3}	3.11×10^{-4}	3.23×10^{-3}	3.00×10^{-4}	6.44×10^{-5}	4.64×10^{-4}	3.08×10^{-5}	1.32×10^{-3}
**FE kg P eq**	1.98×10^{-4}	2.51×10^{-5}	1.78×10^{-5}	4.12×10^{-5}	6.97×10^{-6}	4.38×10^{-5}	9.18×10^{-7}	6.19×10^{-5}
**RU-f MJ**	8.22	5.16×10^{-1}	1.15	8.38×10^{-1}	1.25×10^{-1}	2.02	1.60×10^{-1}	3.41
**RU-mm kg Sb eq**	1.35×10^{-5}	3.35×10^{-6}	4.21×10^{-6}	1.51×10^{-6}	6.73×10^{-7}	2.14×10^{-6}	3.86×10^{-7}	1.20×10^{-6}

Table S4. LCIA results for the degradation process of 1 m^3 of water contaminated with 1 mg/L of caffeine, at pH 5.

pH 5.0 - 1 mg/L of caffeine							
	**Total**	**Plant**	**H_2SO_4**	**$FeCl_3$**	**H_2O_2**	**Transport chemicals**	**Electricity**
**CC kg CO_2 eq**	2.92	3.85×10^{-1}	1.54×10^{-2}	8.54×10^{-3}	1.16×10^{-1}	1.07×10^{-2}	2.39
**OD kg CFC-11 eq**	3.38×10^{-7}	5.94×10^{-8}	1.37×10^{-9}	3.13×10^{-9}	1.39×10^{-8}	2.37×10^{-9}	2.58×10^{-7}
**HT-nc CTUh**	8.62×10^{-8}	4.74×10^{-8}	7.95×10^{-10}	3.88×10^{-10}	1.39×10^{-9}	1.37×10^{-10}	3.61×10^{-8}
**HT-c CTUh**	1.62×10^{-8}	1.47×10^{-8}	2.80×10^{-11}	1.15×10^{-11}	2.95×10^{-10}	4.14×10^{-12}	1.11×10^{-9}
**A mol H+ eq**	2.84×10^{-2}	4.56×10^{-3}	1.98×10^{-3}	6.44×10^{-5}	4.64×10^{-4}	3.08×10^{-5}	2.13×10^{-2}
**FE kg P eq**	1.43×10^{-3}	3.68×10^{-4}	1.09×10^{-5}	6.97×10^{-6}	4.38×10^{-5}	9.18×10^{-7}	9.97×10^{-4}
**RU-f MJ**	6.54×10^{1}	7.56	7.08×10^{-1}	1.25×10^{-1}	2.02	1.60×10^{-1}	5.48×10^{1}
**RU-mm kg Sb eq**	7.42×10^{-5}	4.91×10^{-5}	2.58×10^{-6}	6.73×10^{-7}	2.14×10^{-6}	3.86×10^{-7}	1.93×10^{-5}

Table S5. LCIA results for the degradation process of 1 m^3 of water contaminated with 50 mg/L of caffeine, at pH 2.8.

pH 2.8 - 50 mg/L of caffeine								
	**Total**	**Plant**	**H_2SO_4**	**NaOH**	**$FeCl_3$**	**H_2O_2**	**Transport chemicals**	**Electricity**
**CC kg CO_2 eq**	1.61	1.95×10^{-1}	2.38×10^{-2}	4.80×10^{-2}	8.54×10^{-3}	1.16×10^{-1}	1.07×10^{-2}	1.21
**OD kg CFC-11 eq**	2.29×10^{-7}	3.02×10^{-8}	2.13×10^{-9}	4.61×10^{-8}	3.13×10^{-9}	1.39×10^{-8}	2.37×10^{-9}	1.31×10^{-7}
**HT-nc CTUh**	4.66×10^{-8}	2.41×10^{-8}	1.23×10^{-9}	1.04×10^{-9}	3.88×10^{-10}	1.39×10^{-9}	1.37×10^{-10}	1.83×10^{-8}
**HT-c CTUh**	8.43×10^{-9}	7.48×10^{-9}	4.34×10^{-11}	3.23×10^{-11}	1.15×10^{-11}	2.95×10^{-10}	4.14×10^{-12}	5.64×10^{-10}
**A mol H+ eq**	1.70×10^{-2}	2.32×10^{-3}	3.06×10^{-3}	3.04×10^{-4}	6.44×10^{-5}	4.64×10^{-4}	3.08×10^{-5}	1.08×10^{-2}
**FE kg P eq**	8.03×10^{-4}	1.87×10^{-4}	1.69×10^{-5}	4.18×10^{-5}	6.97×10^{-6}	4.38×10^{-5}	9.18×10^{-7}	5.05×10^{-4}
**RU-f MJ**	3.59×10^{1}	3.84	1.10	8.50×10^{-1}	1.25×10^{-1}	2.02	1.60×10^{-1}	2.78×10^{1}
**RU-mm kg Sb eq**	4.35×10^{-5}	2.49×10^{-5}	3.99×10^{-6}	1.53×10^{-6}	6.73×10^{-7}	2.14×10^{-6}	3.86×10^{-7}	9.79×10^{-6}
